# Effect of Transcranial Pulsed Current Stimulation on Fatigue Delay after Medium-Intensity Training

**DOI:** 10.3390/ijerph19127042

**Published:** 2022-06-08

**Authors:** Qingchang Wu, Guoliang Fang, Jiexiu Zhao, Jian Liu

**Affiliations:** 1 College of Sports Science, Nantong University, Nantong 226019, China; 2015310010@stmail.ntu.edu.cn; 2 China Institute of Sport Science, Beijing 100061, China; fangguoliang@ciss.cn (G.F.); zhaojiexiu@ciss.cn (J.Z.)

**Keywords:** transcranial pulsed current stimulation, medium-intensity exercise, heart rate variability, fatigue, physical function, physiological state, exercise ability

## Abstract

The purpose of this study was to investigate the effect of transcranial pulsed current stimulation (tPCS) on fatigue delay after medium-intensity training. Materials and Methods: Ninety healthy college athletes were randomly divided into an experimental group (*n* = 45) and control group (*n* = 45). The experimental group received medium-intensity training for a week. After each training, the experimental group received true stimulation of tPCS (continuous 15 min 1.5 mA current intensity stimulation). The control group received sham stimulation. The physiological and biochemical indicators of participants were tested before and after the experiment, and finally 30 participants in each group were included for data analysis. Results: In the experimental group, creatine kinase (CK), cortisol (C), time-domain heart rate variability indices root mean square of the successive differences (RMSSD), standard deviation of normal R-R intervals (SDNN), and frequency domain indicator low frequency (LF) all increased slowly after the intervention. Among these, CK, C, and SDNN values were significantly lower than those in the control group (*p* < 0.05). Testosterone (T), T/C, and heart rate variability frequency domain indicator high frequency (HF) in the experimental group decreased slowly after the intervention, and the HF value was significantly lower than that in the control group (*p* < 0.05). The changes in all of the indicators in the experimental group were smaller than those in the control group. Conclusion: The application of tPCS after medium-intensity training enhanced the adaptability to training and had a significant effect on the maintenance of physiological state. The application of tPCS can significantly promote the recovery of autonomic nervous system function, enhance the regulation of parasympathetic nerves, and delay the occurrence of fatigue.

## 1. Introduction

Fatigue often accompanies high-load exercise, and long-term high-load exercise will prolong the elimination time of fatigue, eventually leading to decline in athletic capacity and increased risk of athletic injury [1,2]. Therefore, how to maintain the motor function of athletes, accelerate the elimination of fatigue, and improve sport ability is a primary focus of sports science research. Transcranial electrical stimulation (TES) is a new method to maintain athlete’s physical function and improve exercise capacity, including transcranial direct current stimulation (tDCS), transcranial pulsed current stimulation (tPCS), and transcranial alternating current stimulation(tACS), among which tDCS has been widely used [3,4,5]. Cogiamanian et al. found that using tDCS stimulation increases muscle endurance and prolongs fatigue time [6]. Donati and Park et al. proved that tDCS stimulation technology can enhance physical function of athletes and improve their motor ability by regulating the excitability of the cerebral cortex [7,8]. When fatigue occurs, the discharge frequency of neurons in the brain decreases, and the activation of neuronal activity is insufficient [9]. By inducing net direct current component and the on/off nature of pulsatile currents, tPCS produces tonic effect and phase effect, which improves the excitability of neurons The depolarization process of cell membranes occurs repeatedly with the transmission of each pulse, causing the depolarization process to produce a cumulative effect, and finally produce greater changes in cortical excitability [10]. It is found that tDCS research is the most, while tPCS research is relatively few. At present, most of the research on the impact of transcranial pulse electricity on brain function focuses on basic research, and few studies focus on practical application [11,12,13]. At present, only a few studies involve sports and cognitive function [14,15], while the research on common athlete fatigue is in a blank state. In contrast, tDCS has carried out a lot of research in this field and achieved remarkable results [2,16,17], this makes it possible for tPCS research belonging to TES to have a large research space. Additionally, due to the current characteristics of bipolar pulse of tPCS, it will not have the same electrochemical effect and side effects as tDCS. Therefore, tPCS may have a greater effect on fatigue, which makes this study of high value, so our team conducted this study.

Testosterone (T), cortisol (C), and creatine kinase (CK) in blood are indicators reflecting individual physiological state. Among them, T and C have long been studied as potential biomarkers of catabolic or anabolic state of the body, because they are closely related to the regulation of protein decomposition and synthesis [18]. Creatine kinase (CK) means muscle damage [19]. At present, these indicators reflecting individual physiological states have been used by many scholars to represent individual fatigue states [1,20]. As a non-invasive monitoring method, heart rate variability (HRV) has been widely used in fatigue monitoring [21]. Therefore, in this study, we aimed to assess whether tPCS can affect athletes’ physiological states and fatigue by observing the values of T, C, and CK in the blood and HRV after medium-intensity training. Our research findings will be helpful to provide a new method for faster and more effective physiological recovery after sports training and provide experimental support for the practical application of tPCS. The hypothesis of this study was as follows: after sports training, tPCS can reduces the concentration of CK and C in the blood, improve the overall level of HRV, and effectively help athletes recover their physiological state and delay sports fatigue [15,22].

## 2. Participants and Methods

### 2.1. Ethics

All of the participants signed the informed consent form. The study was carried out in line with the Declaration of Helsinki and was approved by the Institutional Ethics Committee (16 November 2020 (no. 1) by Nantong University).

### 2.2. Participants

**Figure 1:**
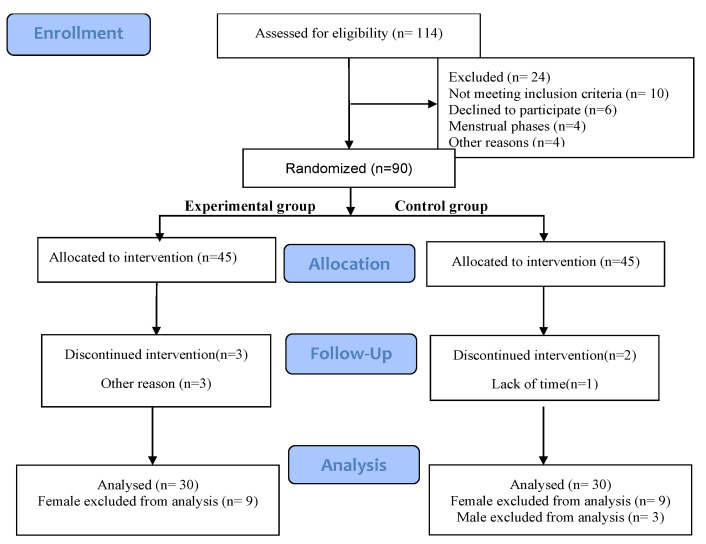
CONSORT Flow Diagram.

Ninety healthy college athletes were selected as participants and randomly divided into an experimental group and control group. Finally, 30 people in each group (all male) participated in the whole process of the experiment (Table 1, Figure 1). All of the athletes who volunteered to participate in this study were in good physical condition, with no cardiovascular system and other diseases, no injuries in the previous 4 weeks, and no physical exercise in the one week before the experiment. During the experiment, the athletes did not participate in any activities that could lead to physical functional decline (e.g., overtraining) or increase (e.g., massage, physiotherapy). Before the test, participants received an explanation regarding transcranial pulsed electrical stimulation (tPCS), the intention of the experiment, and the experimental procedure.

### 2.3. Study Design

This is a randomized double-blind experiment. Subjects are all from the national standard secondary track and field athletes in Nantong University. They voluntarily participated in this experiment. After screening, 90 subjects were obtained. We divided the subjects into two groups with a ratio of 1:1. Using the scale method in simple randomization, the odd number is the experimental group and the even number is the control group and after multiple assignments, 45 subjects in each group. During the experiment, in order to ensure the concealment of distribution, tPCS was managed by personnel who could operate but did not know the intention and process of this study, and the subjects did not know the grouping and stimulation type.

Ninety participants were randomly divided into two groups, with fourty-five participants in each group, and they received true or sham (controls) stimulation of tPCS. In this study, we referred to the research paradigm of Yang et al. on TES stimulation parameters [23]. The stimulation time of tPCS was set to 15 min and the stimulation intensity was set to 1.5 mA. In order to ensure the safety of the experiment, all of the operations were conducted in accordance with the Copenhagen Consensus [24]. First, a 5 × 9 cm^2^ electrode was placed in the central supraorbital region of the frontal lobe, and two 5 × 5 cm^2^ electrodes were placed near the mastoid process of the left and right ear, respectively, and fixed with bandages. During true stimulation, the operator increased the current from 0 mA to 1.5 mA within 30 s. The sham stimulation group received the same stimulation time, stimulation intensity, and current acceleration process, but the current intensity was adjusted to 0 mA 30 s after the initial acceleration. All of the participants were kept quiet during the stimulation to avoid irrelevant stimuli. During the whole process of intervention, if there were adverse reactions (pain or vertigo), the experiment was stopped immediately. At the end of each stimulation, the participants completed a stimulus perception questionnaire to record their feelings.

### 2.4. Experiment Flow

Each participant underwent venous blood extraction and HRV testing the day before and the morning after the experiment. The day before the experiment, participants were taken by the team leader to the laboratory. Participants rested in the sitting position for 5 min before the experiment. The person in charge of each participant explained the experimental process, purpose, and matters needing attention to the participant, and staff input the basic information of participants and used Firstbeat Sports HRV to collect the signals from participants to ensure synchronization of the HRV index and routine biochemical index. Blood was collected from the left antecubital vein immediately after the HRV test. This procedure was carried out the day before the experiment and after the experiment. RPE test was conducted before formal training, tPCS intervention was conducted immediately after formal training, followed by RPE test. Such operation lasted for 7 days.

### 2.5. Training Protocols

All of the participants adopted the medium-intensity training standard recommended by the American College of Sports Medicine (heart rate 140–150 beats per minute) [25]. The athletes engaged in training for one week (7 days). Physical training tasks were carried out on the first, third, fifth, and seventh days, mainly 1500 m and 400 m track and field training. Technical and tactical training tasks were carried out on the second, fourth, and sixth days, mainly to train in special technical movements. Training was held for 3 h a day. The intensity was maintained at a heart rate of 140–150 bpm during the main training time, and the Borg score was more than 8 points after training (Table 2). The specific training protocols are as follows.

Before the training, the participants were familiar with the RPE scale. The exercise trainer supervised the training and assessed the subjects’ exertion from the RPE scale and heart rate.

The subjects began training at 3 p.m. every day. All subjects wore Firstbeat sports to monitor their heart rate and collect their RPE baseline before training. Then, each subject performed warm-up exercises, such as leg pressing exercises, leg kicking exercises, stretching of low back muscles, and then jogging training. After the warm-up action, they conducted a warm-up run of 5 laps exercise, and then started formal training.

Physical training was conducted on the first, third, fifth, and seventh days. The arrangement of physical training was as follows: after the auxiliary training, repeat the 4000 m long-distance run 6 times. The six long-distance runs were divided equally into two sections. There was no rest in the first section, but 15 min rest between the two sections. The rest time of the last three long-distance run was 3 min and 5 min, respectively. After the training, the RPE value was recorded again.

Technical and tactical training was conducted on the second, fourth, and sixth days. When technical and tactical training was conducted, all auxiliary training was consistent with physical training. Then, the coach asked the subjects to carry out technical and tactical training.

Explosive power training: repeat the 400 m training for 10 times, with an interval of 2 min between each training.

Then, we did relaxation training.

Then, general physical fitness exercises were carried out, such as 40 sit ups, 40 push ups, and 50 m acceleration runs for 3–4 times.

Then, leg pressing, kicking exercises, etc.

Then, endurance training: repeat the 1500 m training for 7 times, with an interval of 3 min between each training, correct the technical action at the same time, and record the RPE value again after the training.

### 2.6. Experimental Equipment

#### 2.6.1. Transcranial Pulsed Current Stimulation

Developed by National Key Technology R&D Program of China, the stimulation current is a bipolar current of 60–80 Hz, the pulse waveform is a square wave, the duty cycle is 29.7%, the stimulation intensity is in the range of 0–2 mA, and the stimulation time is determined by the experimental program. This product passed national security certification on 17 April 2021: report number CHTSM21040049.

#### 2.6.2. HRV Test

Related indicators of HRV tested using an HRV tester made in Finland (model: Firstbeat Sports). RR-interval Measurement: BG3 measures beat-by-beat intervals as time between consecutive R-peaks in ECG. Measurement resolution: 1 ms.

#### 2.6.3. Blood Collection

We used 5 mL EDTA-anticoagulated vacutainer tubes to collect blood samples from the participants, which were produced by Lingen Precision Medical Products (Shanghai) Co., Ltd. (Shanghai, China).

#### 2.6.4. Questionnaire for Rated Perceived Exertion (RPE)

The RPE score was 5–7 in moderate exertion and 8–10 in severe exertion. The reliability of assessment of fatigue degree of the RPE has been verified, and the correlation coefficient between groups is 0.71–0.90 [26,27].

### 2.7. Physiological State Index Test

Physiological state indicators included T, C, T/C, and CK. HRV included time-domain indicators: the standard deviation of normal R-R intervals (SDNN) and root mean square of the successive differences (RMSSD). Frequency domain indicators included high frequency (HF) and low frequency (LF) [20,28,29,30,31].

### 2.8. Sample Size and Randomization

In this study, the sample size is based on a two-sided t-test. We set the type I error probability of hypothesis test α is 0.1, and the type II error rate of hypothesis test β is 0.2, considering a 1:1 allocation rate and a drop-out rate of 20%. After calculation and looking up the table, we recruited a total of 114 patients for two groups. Additionally, we found that most of the subjects in similar studies were 30 [15,32]; therefore, the 60 subjects meet the experimental requirements.

### 2.9. Data Processing and Analysis

In this experiment, blood was collected twice, the day before and the day after the experiment. After the subjects reached the blood collection room (the temperature is 20 °C), they sat quietly and rested for 10 min, and then the medical staff took 4 ml of left elbow venous blood and collected the blood sample in a vacuum blank tube to avoid shaking and vibration of the contents (blood).Within 30 min, serum was separated using a high-speed centrifuge (2000 R/min, 15 min. Shu Ke, China). The supernatant was extracted and stored in a medical refrigerator at −80 °C (Boko, BDF-86V158, China). CK, T, and C were detected using ELISA kits (Wuhan Jianglai Biotechnology Co., Ltd. (Wuhan, China), according to the manufacturer’s instructions, and the whole process was supervised by a principal investigator. All samples were sent to the laboratory for biochemical analysis within 48 h, and all analyses were repeated and performed by trained technicians. Serum samples were analyzed using an automatic biochemical analyzer (RaytoRT-6100 China). Serum CK, T, and C levels were recorded, and the serum T/C ratio was calculated. With abnormal fluctuations, interference, and pseudo-errors were eliminated using Polar software. Five-minute signal samples were selected for HRV time domain and frequency domain analysis.

Statistical analysis was carried out using IBM SPSS 22.0 (IBM Corp., Armonk, NY, USA) and Microsoft Excel 2019 (Microsoft Corporation, Redmond, WA, USA). The obtained parameters are expressed as mean ± standard deviation. The Shapiro–Wilk test was used to examine the normal distribution of the outcomes. Repeated measures analysis of variance (ANOVA) was used to analyze the effects of each variable (group × measurement time) on each index of function and each index of HRV. Then, post hoc analysis was used if a significance in the interaction was observed and corrected using Bonferroni correction. The significance level was set at *p* < 0.05 and the highest significance level was set at *p* < 0.01.

## 3. Results

In this study, 60 participants completed the entire experimental process. Before the experiment, there were no significant differences between the two groups in the homogeneity test for each physical functional state index (Table 3).

### 3.1. Changes in Blood Marker Indicators after tPCS Intervention

**Figure 2:**
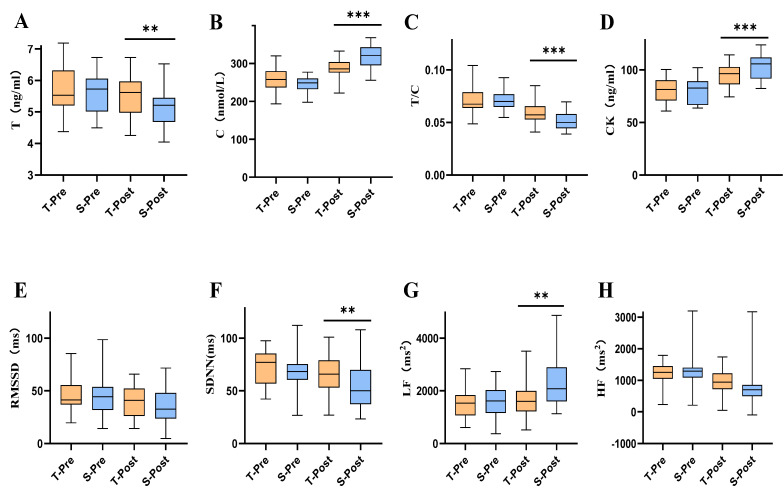
Effects of tPCS intervention programs on indicators of blood markers and HVR after medium−intensity training. Note: (**A**): Effects of tPCS intervention on T; (**B**): Effects of tPCS intervention on C; (**C**): Effects of tPCS intervention on T/C; (**D**): Effects of tPCS intervention on CK; (**E**): Effects of tPCS intervention on RMSSD; (**F**): Effects of tPCS intervention on SDNN; (**G**): Effects of tPCS intervention on LF; (**H**): Effects of tPCS intervention on HF. ** represents *p* < 0.05; *** represents *p* < 0.01; Orange (◼) means experimental group; Blue (◼) means control group (the same below). T−pre means before true intervention; S−pre means before sham intervention; T−post means after true intervention; S−post means after sham intervention.

**Figure 3:**
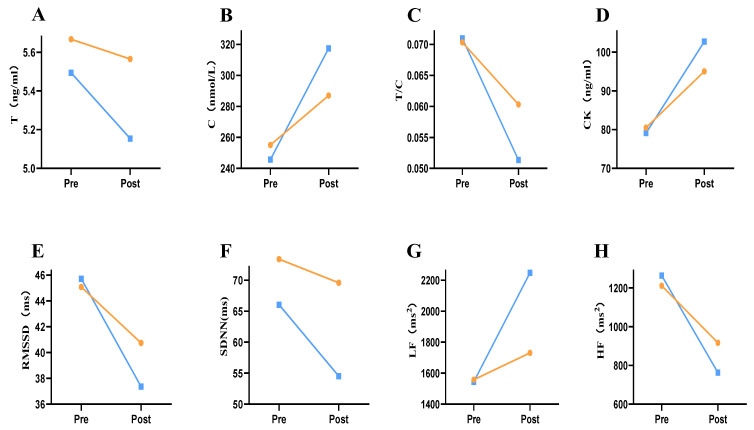
Changes in blood marker values and HRV values before and after tPCS intervention. Note: (**A**): Changes in T values before and after tPCS intervention; (**B**): Changes in C values before and after tPCS intervention; (**C**): Changes in T/C values before and after tPCS intervention; (**D**): Changes in CK values before and after tPCS intervention; (**E**): Changes in RMSSD values before and after tPCS intervention; (**F**): Changes in SDNNs value before and after tPCS intervention; (**G**): Effects of tPCS intervention on LF; (**H**): Changes in HF values before and after tPCS intervention. Pre means before intervention; Post means after intervention.

The effects of group, measurement time, and their interaction on T, C, T/C, and CK were analyzed by ANOVA. The results showed that C [F _(1, 58)_ = 14.478077, *p* < 0.001], T/C [F _(1, 58)_ = 8.024, *p* = 0.006], and CK [F _(1, 58)_ = 5.171, *p* = 0.027] had the interaction effects of group and measurement time; T [F _(1, 58)_ = 9.330, *p* = 0.003], C [F _(1, 58)_ = 97.966, *p* < 0.001], T/C [F _(1, 58)_ = 76.102, *p* = 0.003], and CK [F _(1, 58)_ = 90.626, *p* < 0.001] had significant time main effects (Table 3). Post hoc comparison showed significant differences in T, C, T/C, and CK values between the two groups; *p* values were P_T_ = 0.028, P_C_ < 0.001, P_T/C_ =0.006, and P_CK_ = 0.027 (Table 4, Figure 2A–D). Both T and T/C values decreased after the intervention, and the decrease in the experimental group was smaller than that in the control group, with the difference being 4.313% and 19.195%, respectively. The values of C and CK increased after intervention, and the increase in the experimental group was less than that in the control group; the difference was 16.700% and 11.848%, respectively (Figure 3A–D).

### 3.2. Changes in HRV Indicators after tPCS Intervention

The effects of group, measurement time, and their interaction on RMSSD, SDNN, LF, and HF were analyzed by ANOVA. Results showed that LF [F _(1, 58)_ = 7.032, *p* = 0.010] and HF [F _(1, 58)_ = 7.410, *p* = 0.009] had the interaction effect of group and measurement time; RMSSD [F _(1,58)_ = 8.254, *p* = 0.006], SDNN [F _(1, 58)_ = 16.366, *p* = 0.000], LF [F _(1, 58)_ = 19.263, *p* < 0.001], and HF [F _(1, 58)_ = 109.850, *p* < 0.001] had significant time main effects (Table 3). Post hoc comparison revealed significant differences in SDNN and LF values between the two groups, with *p* values P_SDNN_ = 0.039 and P_LF_ = 0.01 (Table 4, Figure 2E–H). RMSSD, SDNN, and HF values decreased after the intervention, and the decrease in the experimental group was smaller than that in the control group, with differences being 8.655%, 11.624%, and 15.341%, respectively. The values of LF increased after intervention, and the increase in the experimental group was less than that in the control group; the difference was 34.418% (Figure 3E–H).

## 4. Discussion

In this study, we found that after medium-intensity exercise, testosterone levels in the experimental group and control group decreased by 4% and 8%, respectively, compared with those before the experiment, which means that tPCS intervention after medium-intensity intervention can delay the decrease in the overall testosterone level. Karkoulias et al. [33] found in the marathon monitoring of non-elite athletes that long-term endurance exercise will lead to the decrease in testosterone levels. Frana and others further proved the change law of testosterone during exercise. He found that testosterone increased immediately after medium-intensity training but began to decrease when the exercise time was more than 2 h [34]. A possible reason for this is that short-term inexhaustible exercise can promote the secretion of testosterone [35]. Slivka et al. [36] found that testosterone concentrations decreased continuously among athletes during 21 days of mountain bike cross-country training. Raastad et al. [37] also found that testosterone concentrations decreased significantly after high-intensity strength training; participants were exhausted due to a lot of sugar and protein consumption caused by high-intensity training, resulting in hypothalamic dysfunction, which led to the decrease in testosterone concentration. This is consistent with the results obtained after high-intensity exercise in our study. In this study, testosterone in the experimental group decreased by 4% compared with levels before the experiment, and the decrease in the experimental group was less than that in the control group, indicating that the testosterone concentration in the experimental group was higher than that in the control group after tPCS intervention. The tPCS can stimulate the deep brain nuclei, and the production of testosterone is controlled by the hypothalamus–pituitary–gonad axis (HTPG). Therefore, we speculate that the oscillatory current of tPCS may affect the activity of HTPG and promote the secretion of testosterone, so as to delay the decline in testosterone concentration and finally maintain the individual’s motor function, but this speculation needs further verification [11,38].

After tPCS intervention, the increase in cortisol concentrations in the experimental group was lower than that in the control group. Crew et al. [39] reported that cortisol will physiologically affect sports performance. When cortisol is too high, it will lead to a decline in athletes’ overall sports performance [40]. Cortisol may affect exercise performance physiologically, many scholars have found that a high concentration of cortisol is often associated with low sport performance [41,42]. Numerous studies [43,44,45] have shown that athletes have a significant increase in cortisol after exercise. The research results of Foretic et al. show that cortisol concentrations after exercise were 67% higher than that before exercise. In this study, cortisol concentrations increased significantly after exercise in both the experimental group and control group. Cortisol in the control group increased by 29.2% and the experimental group increased by 12.5% after exercise. The increase of cortisol in the experimental group was less than that in the control group, which was consistent with the study of Mehrsafar et al. [46]. Those authors found that when athletes participate in high-intensity competition, the use of tDCS in the dorsolateral prefrontal cortex (DLPFC) can reduce the cortisol concentration in athletes. Similarly, the results of Brunoni [22] showed that when the anode of tDCS was used to stimulate the DLPFC in 20 healthy adults, the concentration of cortisol could be reduced, which was consistent with changes in athletes’ frontal lobe stimulated by tPCS in this study. Some studies have also pointed out [47] that when using tDCS to stimulate the brain of C3 position (Brain localization based on 10–20 EEG system), the concentration of cortisol will decrease significantly, with a decrease range of 24.18%. tPCS and tDCS mentioned above belong to TES [48] and are highly similar in function and use. It can be speculated that the stimulation of the frontal lobe by tPCS may lead to top-down regulation and finally affects the regulation of the hypothalamic–pituitary–adrenal axis, thereby reducing the concentration of cortisol; however, the specific mechanism remains to be further verified. In this study, after one week of tPCS intervention, the decline in T/C values in the experimental group was 19.195% less than that in the control group, indicating that tPCS intervention can maintain the function of exercise and delay the accumulation of fatigue. We speculate that the reason is that tPCS can activate brain neurons, promote T synthesis, and inhibit cortisol secretion, avoiding loss of muscle strength and the decline in individual function; the action path needs to be further studied.

Feng et al. [49] found that the creatine kinase value reflects the stress degree of the body on exercise load in the stage of medium-intensity exercise and reflects the recovery of the body after exercise. Through intervention with tPCS in medium-intensity training, we found that the increase in creatine kinase in the experimental group after the intervention was 11.848%, which was lower than that in the control group. This means that the physical state of participants in the experimental group was better than that in the control group. Additionally, we found that after one week of tPCS intervention, college athletes in the experimental group recovered better than those in the control group. The increase in creatine kinase values in the experimental group was less than that in the control group, which was consistent with a study by Chen et al. [50], who concluded in the blood analysis among fencers that when athletes undergo physical fitness and special training, creatine kinase values will rise significantly, and the degree of fatigue is high. After a week of adjustment and training, the creatine kinase value of athletes drops to the lowest point, at this time, they are in good physical condition. This may create good conditions for the recovery of athletes’ muscle damage at this time, which provides a premise for athletes to return to the competition as soon as possible. Therefore, we speculate that the application of tPCS after medium-intensity training is helpful to the recovery of the athletes’ physical states.

The HRV SDNN indicator mainly reflects the tension in the sympathetic nerve and vagus nerve, which is used to evaluate the overall degree of autonomic nerve damage and recovery. In this study, compared with the experimental group, the SDNN in the control group decreased significantly (*p* < 0.05). However, athletes in the experimental group maintained their state due to tPCS intervention, so the change in SDNN was not obvious. This shows that after medium-intensity training, athletes’ function is decreased, the fatigue state appears, and the SDNN index value decreases significantly. The SDNN changed little after tPCS intervention, and there was no significant difference before and after intervention. However, the findings of Zeng et al. [51] differed from those of our study. That study showed that the occurrence of fatigue will increase the SDNN value in driving tasks. We speculate that this may be related to different fatigue states. Zeng et al. conducted a mental fatigue model of a vigilance task; in this study, we carried out an intervention of a sports fatigue model, and the fatigue state reached exhaustion. Therefore, there were inconsistent changes in SDNN indicators. Some scholars [52,53] have pointed out that SDNN decreases significantly when tDCS stimulation is performed before exercise, which is different from our study. The reason may be that tDCS stimulation breaks the sympathetic/parasympathetic balance before exercise. This suggests that we should pay attention to the timing of intervention when applying tPCS and the specific application scheme needs to be further explored.

Some scholars [54] have found that the RMSSD is highly correlated with physical activity and fatigue, the higher the RMSSD value, the lower the fatigue level. Haddad further stated that RMSSD is the most reliable HRV index [55]. In this study, the RMSSD index of the experimental group was 8.655% lower than that of the control group, indicating that the degree of fatigue could be delayed after tPCS intervention. Garet et al. [56] found that the HRV index decreased by 22% after 3 weeks of overload training in swimmers, whereas Pichot et al. [57] found that the HRV indicator decreased by 38% after one week of overload training among long-distance runners. After reducing the training intensity and subsequently training for two weeks, the HRV indicator in swimmers increased by 7% and that of long-distance runners increased by 38%, which is consistent with the results of this study. When athletes in the control group had a week of high-intensity exercise, SDNN reflecting the overall level of HRV and RMSDD reflecting the activity of parasympathetic nerves decreased significantly by 22.64% and 18.27%, respectively. Although the SDNN and RMSSD indexes of the experimental group also decreased, they were both lower than those of the control group. SDNN was 11.62% lower than the control group, and RMSSD was 8.66% lower than the control group. This shows that although athletes were in the state of fatigue, the activity level of parasympathetic nerves in the experimental group was higher, which can reduce the degree of fatigue and enable athletes to have better sports performance [58]. However, the lower parasympathetic activity in the control group indicates poor recovery conditions after exercise or being prone to fatigue.

Both the sympathetic and parasympathetic nervous systems contribute to the LF power peak (0.04–0.15 Hz). LF reflects overall sympathetic and parasympathetic activity [59]. After tPCS intervention, the LF value in the experimental group increased less than that in the control group. Uusitalo [59] found that when athletes overtrained for 6–9 weeks, there was a significant increase in LF indicators. Montenegro et al. [60] applied tDCS to the left temporal region of professional road cyclists and found that LF decreased and HF increased during competition; this is consistent with the results of this study. We found that the HRV frequency domain indicator LF in the experimental group had no significant change before and after the intervention, whereas the LF indicator in the control group increased significantly due to training (*p* < 0.05). Further research by Montenegro found that this change was only seen in professional road cyclists but not in non-athletes, which may be due to higher neural efficiency in the central command of the autonomic nervous system and faster recovery from exercise after tDCS stimulation [61]. To a certain extent, this shows that tPCS intervention has an inhibitory effect on the decrease in athletes’ function or a positive effect on the maintenance of physical function, which can prevent fatigue and diminished sports performance caused by the rapid decline in function during competition. The significant increase in LF indicators in the control group indicates the enhancement of sympathetic nerve activity and the inhibition of the recovery of individual function.

The contribution of parasympathetic nerve activity mainly appears in the HF power peak (0.15–0.40 Hz), and HF mainly reflects parasympathetic nerve activity. Kliszczewicz et al. [62] found that HF values decrease significantly after high-intensity functional training, which is consistent with the results of our study. Pichon and other scholars [63] reported different findings in their research. When athletes conduct constant load training, LF decreases significantly and HF increases significantly. These research findings suggest that vigorous exercise may lead to a change in participants’ respiratory dynamics and affect the balance of sympathetic/parasympathetic nerve tension [64]. We found that the HF index of the experimental group and control group decreased before and after tPCS intervention, with 24.33% in the experimental group and 39.64% in the control group. This shows that the effect of exercise on HF is significant, the recovery time of HF to baseline is long, and the effect of tPCS intervention on HF is less than exercise.

In this study, we found that the effect of TES on post-training HRV is complex, and attention should be paid to study participants’ state when explaining motor energy and fatigue state. The participants’ physical state, training intensity, training method, mental state, measuring posture, and measuring time can all affect changes in HRV, which lead to differences in results [65,66,67,68]. Fatigue is a process of continuous accumulation. The change in HRV indicators is clear only when exercise accumulates to exhaustion. If athletes monitor the process of fatigue accumulation, HRV indicators may change inversely owing to the activation of the exercise state. When exercise function is low or fatigue occurs, the SDNN index of HRV decreases, and the LF index increases. However, due to the enhancement of exercise intention, the degree of body activation is at a high level, the SDNN index of HRV increases, and the LF index decreases. Although fatigue still exists and is accumulating at this time, it is covered by the activation state of the body, so the change in HRV indicators is not uniform, which has been observed by many scholars [69]. The above research shows that when using HRV to analyze the effect of tPCS, we should comprehensively consider the influence of various factors, especially the influence of athletes’ degree of fatigue. Future research should consider the influence of tPCS on HRV in different degrees of fatigue.

Because blood indices were not monitored every day in this experiment, it was impossible to prove whether participants’ cortisol and other blood indices had exceeded their peak or whether these blood indices never reached their peak or even the minimum. Therefore, it was difficult to accurately determine the best time to use tPCS, whether the best effect can be achieved before or during competition. In future research, the frequency of observation of CK, C, and T values can be increased and the best time to use tPCS identified. In addition, there are some individual differences in the application effect of tPCS. The participants in this study were relatively limited. Therefore, more personalized schemes can be explored in the application of tPCS. For different ages and sexes, more accurate intervention programs should be formulated to enhance the intervention effect of tPCS.

In the future, this study may be applied to the field to reduce athletes’ sports injuries caused by fatigue. On the other hand, due to the effect of tPCS on cognition, it may alleviate the widespread cognitive fatigue of workers, such as driving for a long time or processing documents. It can even be used in the field of rehabilitation to alleviate the decline in the effect of rehabilitation training caused by fatigue. It is gratifying that some scholars have been doing research in these fields and have achieved certain results [15,70]. However, this does not mean that the practical application research of tPCS has matured. At present, the application of tPCS is still less than tDCS, which leaves valuable space for further exploration of the scope of practical application of tPCS, and also puts forward higher requirements for our next research study.

## 5. Conclusions

The application of tPCS after medium-intensity training enhanced the adaptability to training and had a significant effect on the maintenance of the physiological state. The application of tPCS can significantly promote the recovery of autonomic nervous system function, enhance the regulation of parasympathetic nerves, and delay the occurrence of fatigue.

## Figures and Tables

**Table 1:** Basic information of participants.

	Experimental Group	Comparison Group	*p*-Value
Age/years	20.47 ± 0.72	20.63 ± 1.46	0.683
Height/cm	177.89 ± 7.24	178.37 ± 6.40	0.832
Weight/kg	73.97 ± 10.99	70.58 ± 8.96	0.315
Training years	4.12 ± 0.99	4.00 ± 0.75	0.688

**Table 2:** Training volume and training time.

Day	Total Training Distance	Main Training Time/min	Auxiliary Training Time/min	Borg
First day	20 km	138 min	42 min	8.53 ± 0.67
Second day	14 km	108 min	72 min	8.26 ± 0.44
Third day	22 km	144 min	36 min	8.80 ± 0.75
Fourth day	16 km	113 min	67 min	8.36 ± 0.60
Fifth day	18 km	124 min	56 min	9.00 ± 0.68
Sixth day	14 km	110 min	70 min	8.66 ± 0.65
Seventh day	18 km	120 min	50 min	8.93 ± 0.72

**Note**: Main training refers to formal running time and auxiliary training refers to technical movement preparation, simulation training, warm-up, and so on.

**Table 3:** Main effect and interactive effects of blood markers and HRV indicators after tPCS intervention.

Indicators		F	*p*	Indicators		F	*p*
T (ng/mL)	Time	9.330	0.003	RMSSD/ms	Time	8.254	0.006
	group	2.813	0.099		group	0.157	0.694
	Time × group	1.535	0.220	SDNN/ms	Time × group	0.828	0.367
C (nmol/L)	Time	97.966	0.000		Time	16.366	0.000
	group	14.084	0.000		group	3.671	0.060
	Time × group	14.478	0.000	LF/ms2	Time × group	1.937	0.169
T/C	Time	76.102	0.003		Time	19.263	0.000
	group	4.445	0.039		group	7.032	0.010
	Time × group	8.024	0.006		Time × group	7.032	0.01
CK (ng/mL)	Time	90.626	0.000	HF/ms2	Time	109.850	0.000
	group	2.393	0.127		group	0.203	0.654
	Time × group	5.171	0.027		Time × group	7.410	0.009

**Table 4:** Analysis of blood markers and HRV results before and after tPCS intervention in the two groups.

Indicators	Test Time	Experimental Group	Control Group	T	*p*	Rate of Change Difference
T (ng/mL)	Pre-training	5.677 ± 0.708	5.565 ± 0.589	0.608	0.545	4.313%
	Post-training	5.494 ± 0.658	5.155 ± 0.552	2.162	0.035	
C (nmol/L)	Pre-training	255.112 ± 31.098	245.701 ± 18.050	1.434	0.157	16.700%
	Post-training	287.021 ± 27.151	317.465 ± 34.937	−3.769	0.000	
T/C	Pre-training	0. 070 ± 0.013	0.071 ± 0.009	−0.228	0.821	13.884%
	Post-training	0.060 ± 0.010	0.051 ± 0.008	3.753	0.000	
CK (ng/mL)	Pre-training	80.509 ± 10.382	79.113 ± 11.745	0.488	0.627	11.848%
	Post-training	95.041 ± 10.811	102.765 ± 11.389	−2.694	0.009	
RMSSD/ms	Pre-training	45.080 ± 14.236	45.716 ± 17.882	0.153	0.879	8.655%
	Post-training	40.744 ± 14.537	37.36 ± 16.73	0.836	0.407	
SDNN/ms	Pre-training	73.399 ± 15.579	69.601 ± 16.900	0.905	0.369	11.624%
	Post-training	66.05 ± 18.010	54.541 ± 23.767	2.114	0.039	
LF/ms^2^	Pre-training	1557.930 ± 590.033	1544.173 ± 579.528	0.091	0.928	34.418%
	Post-training	1731.498 ± 752.877	2247.697 ± 839.009	−2.508	0.015	
HF/ms^2^	Pre-training	1211.880 ± 338.954	1264.589 ± 485.907	−0.487	0.628	15.341%
	Post-training	917.129 ± 400.070	763.014 ± 578.380	1.200	0.235	

**Note**: The rate of change difference refers to the difference between the change rate of each index in the experimental group and control group.
